# Using patient‐specific bolus for pencil beam scanning proton treatment of periorbital disease

**DOI:** 10.1002/acm2.13134

**Published:** 2020-12-24

**Authors:** Minglei Kang, Shaakir Hasan, Robert H. Press, Francis Yu, Mashal Abdo, Weijun Xiong, Jehee I. Choi, Charles B. Simone, Haibo Lin

**Affiliations:** ^1^ New York Proton Center New York NY USA

**Keywords:** bolus, lymphoma, pencil beam scanning, periorbital disease, proton therapy

## Abstract

**Purpose:**

A unique mantle cell lymphoma case with bilateral periorbital disease unresponsive to chemotherapy and with dosimetry not conducive to electron therapy was treated with pencil beam scanning (PBS) proton therapy. This patient presented treatment planning challenges due to the thin target, immediately adjacent organs at risk (OAR), and nonconformal orbital surface anatomy. Therefore, we developed a patient‐specific bolus and hypothesized that it would provide superior setup robustness, dose uniformity and dose conformity.

**Materials/Methods:**

A blue‐wax patient‐specific bolus was generated from the patient's face contour to conform to his face and eliminate air gaps. A relative stopping power ratio (RSP) of 0.972 was measured for the blue‐wax, and the HUs were overridden accordingly in the treatment planning system (TPS). Orthogonal kV images were used for bony alignment and then to ensure positioning of the bolus through fiducial markers attached to the bolus and their contours in TPS. Daily CBCT was used to confirm the position of the bolus in relation to the patient's surface. Dosimetric characteristics were compared between (a) nonbolus, (b) conventional gel bolus and (c) patient‐specific bolus plans. An in‐house developed workflow for assessment of daily treatment dose based on CBCT images was used to evaluate inter‐fraction dose accumulation.

**Results:**

The patient was treated to 24 cobalt gray equivalent (CGE) in 2 CGE daily fractions to the bilateral periorbital skin, constraining at least 50% of each lacrimal gland to under 20 Gy. The bolus increased proton beam range by adding 2–3 energy layers of different fields to help achieve better dose uniformity and adequate dose coverage. In contrast to the plan with conventional gel bolus, dose uniformity was significantly improved with patient‐specific bolus. The global maximum dose was reduced by 7% (from 116% to 109%). The max and mean doses were reduced by 6.0% and 7.7%, respectively, for bilateral retinas, and 3.0% and 13.9% for bilateral lacrimal glands. The max dose of the lens was reduced by 2.1%. The rigid shape, along with lightweight, and smooth fit to the patient face was well tolerated and reported as “very comfortable” by the patient. The daily position accuracy of the bolus was within 1 mm based on IGRT marker alignment. The daily dose accumulation indicates that the target coverage and OAR doses were highly consistent with the planning intention.

**Conclusion:**

Our patient‐specific blue‐wax bolus significantly increased dose uniformity, reduced OAR doses, and maintained consistent setup accuracy compared to conventional bolus. Quality PBS proton treatment for periorbital tumors and similar challenging thin and shallow targets can be achieved using such patient‐specific bolus with robustness on both setup and dosimetry.

## 1 INTRODUCTION

Radiation therapy is widely used for the treatment of orbital tumors.^1^ Periorbital diseases, particularly for skin tumors, are difficult to treat due to the immediately adjacent organs at risk (OARs) of retinas, lens, lacrimal glands, and optic nerves. Traditionally, orthovoltage photon therapy or electron treatments are used to treat superficial lesions. However, due to the challenging anatomy, neither technique currently can provide radiation oncologists with an optimal plan to maintain excellent target coverage while sparing the eye and OARs.^2^ It is well known that proton beam therapy does not have exit dose and the distal dose fall‐off is very sharp, which can potentially offer superior sparing of OARs for periorbital disease.

Proton beams carry charged particles that deposit relatively low doses in the entrance path proximal to the tumor and deposit most of their energy at the end of its path, called the Bragg peak. The depth of the Bragg peak in tissue is determined by the energy of the beam, optimizing dose delivered to the tumor while the OARs beyond the tumor receive very little dose.^3^, ^4^ Proton therapy has shown its potential advantage in the treatment of different kinds of cancer.^5^, ^6^, ^7^, ^8^, ^9^, ^10^, ^11^ Many studies have demonstrated that proton therapy can achieve better OAR sparing and lead to fewer side effects and/or better quality of life preservation than conventional photon radiation.^12^, ^13^, ^14^, ^15^ A recent study reported proton double scattering treatment for intraocular tumors with good efficacy.^16^ Proton pencil beam scanning (PBS) is the latest form of proton radiation treatment,^12^, ^13^, ^14^, ^15^, ^17^ and the advances in PBS technology can benefit periorbital disease as well.

Using PBS proton to treat periorbital disease, treatment planning presents several challenges due to the superficial and very thin target and the immediately adjacent OARs, including the retinas and lacrimal glands. In order to improve the target dose uniformity, gel‐like skin bolus was used to add an additional 1‐cm buildup to accommodate additional proton spot layers, while the nonconformal orbital surface anatomy introduced air gap between the bolus and skin that negated the purpose of the bolus and caused dose uncertainties. Therefore, we designed a patient‐specific blue‐wax bolus and hypothesized that it can provide improved setup consistency, dose uniformity, and dose conformity. Both et al.^18^ implemented a universal U‐shape bolus by replacing range shifter to maintain a small spot size for base of skull PBS applications, Michiels et al.^19^ studied the patient‐specific bolus in preserving PBS spot size for oropharyngeal cancer intensity modulated proton therapy (IMPT). This study aims to present the first treatment experience using a patient‐specific bolus to maximize the PBS proton advantage in treating superficial and extremely thin periorbital disease at our center.

## 2 MATERIALS AND METHODS

**FIG. 1:**
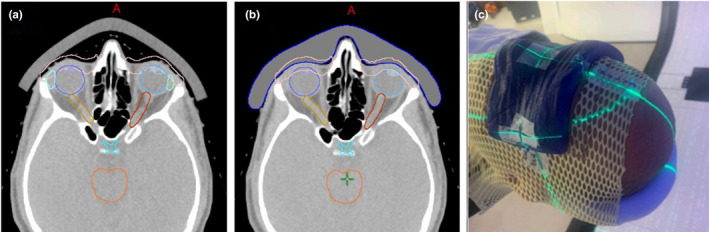
Blue‐wax bolus vs. conventional bolus. (a) the conventional bolus with air gaps; (b) blue‐wax bolus eliminating the air gaps; (c) patient treatment with the blue‐wax bolus on.

**FIG. 2:**
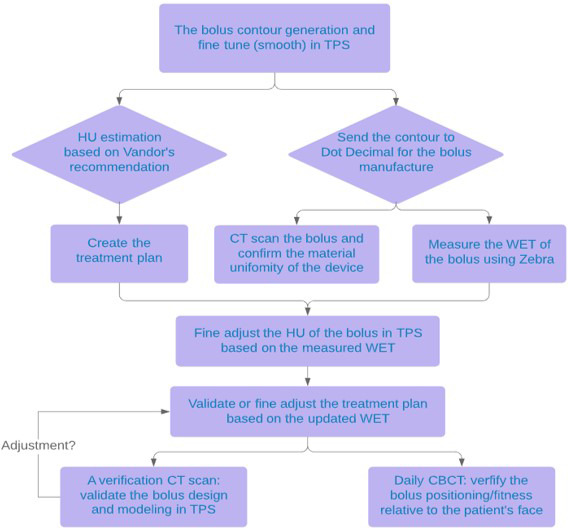
The workflow chart to design, measure and validate the use of bolus for proton therapy.

A healthy 50‐year‐old male with stage IV mantle cell lymphoma was treated with chemotherapy with near complete response except for residual disease in the bilateral periorbital tissue. The residual disease was unresponsive to second‐line chemotherapy and was ultimately treated with PBS proton therapy. Both photons and electrons were considered as well, but the deep penetration of the photon beam and inhomogeneity of electrons, with a global maximum dose of 140%, made them inferior plans. The bilateral periorbital disease had the largest discrete lesion measuring 6 mm wide by 5 mm thick in the right upper eyelid. The patient was treated with a prescription of 24 CGE in two CGE daily fractions to the bilateral periorbital skin, with a dose constraint of at least 50% of each lacrimal gland under 20 Gy.^20^ The depth expansion of the target was only ~5 mm, which needs 2–3 energy layers to deliver the desired dose treatment area (layer spacing ~2.5 sigma of Bragg peak width for the ProBeam system, Varian Medical Systems, Palo Alto, CA). Although a 3‐cm range shifter pulls the proton dose back all the way to the skin, different spot spacing, layer spacing and beam arrangement were optimized, it remains difficult to achieve a uniform dose with adequate target coverage to such a thin target. Bolus was proposed to add more expansion to the target and especially to allow more proton energy layers at the proximal end of the target for dose optimization. Conventional commercially available boluses usually are made of gel‐like materials with relatively uniform density and are widely used in photon therapy for boosting the surface/skin dose. To test its efficacy, a piece of gel bolus was placed over the target area during CT simulation. Even though the bolus was soft and falls onto the patient skin surface with gravity, large air gaps were still introduced to the skin that were more susceptible to proton range and dose uncertainties due to poor reproducibility of daily treatment setup [see Fig. 1(a)]. To eliminate the air gap and reduce uncertainties, we generated a bolus contour conforming to the patient face, which removed all of the air gaps. Polyethylene wax, or blue‐wax, is one of the most popular materials used for proton compensators. The relative stopping power ratio (RSP) of the blue‐wax was measured to be 0.972 with clinical proton beams, and the HUs were overridden appropriately to match the measured value in the treatment planning system (TPS). As shown in Fig. 2, a patient‐specific bolus workflow including bolus design, RSP verification and positioning for treatment was used. The bolus contour was first designed and smoothed in the TPS, then the digital 3D data were sent to the vendor for manufacturing. The vendor used a milling machine to cut a whole piece of blue‐wax to ensure the uniformity of the density of the bolus. The RSP was verified by both CT scanning and Zebra range measurement. The HU of the bolus would then be subject to fine adjustments based on the RSP verification results.

**FIG. 3:**
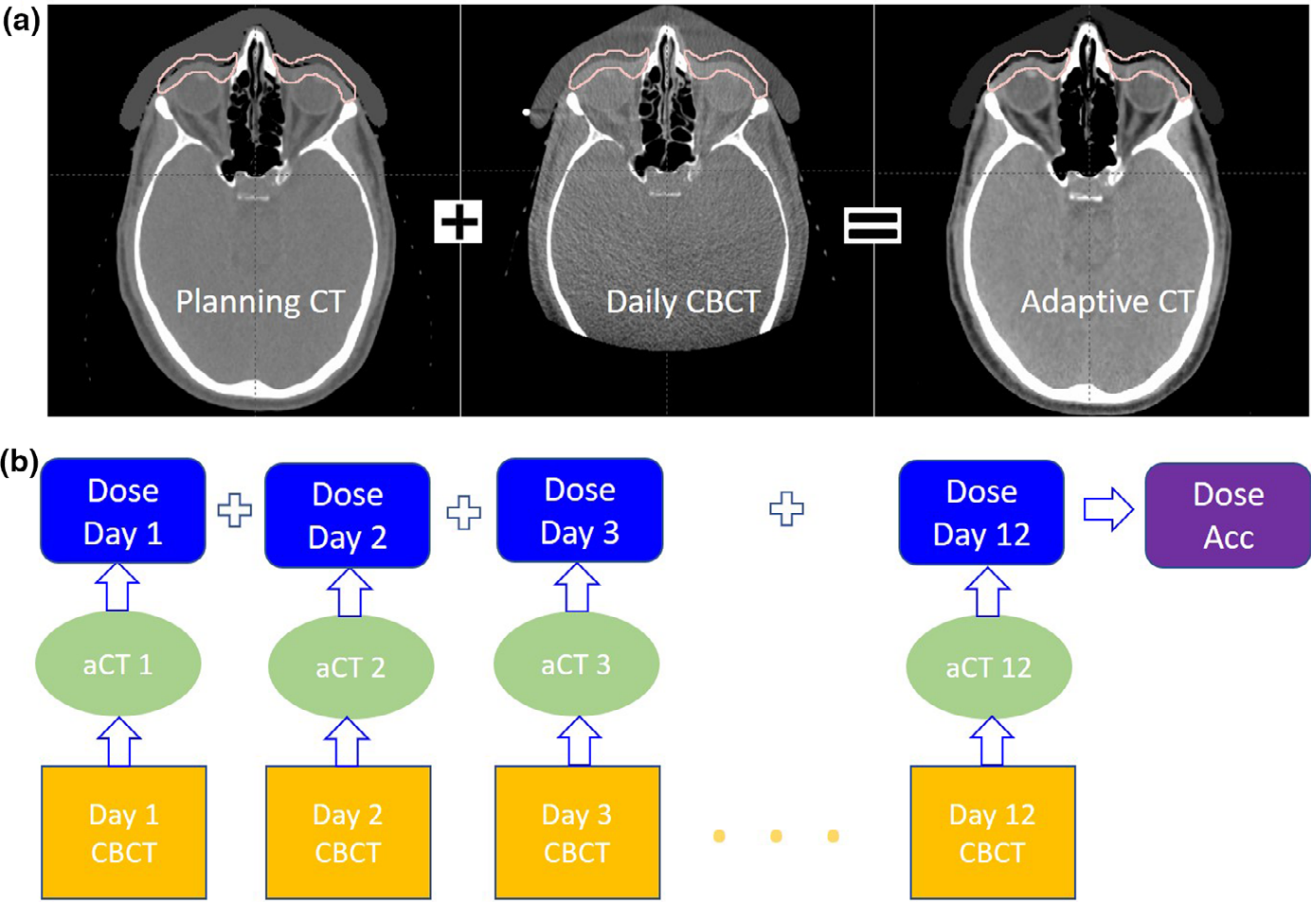
Daily CBCT images were used for daily dose verification and dose accumulation: (a) planning CT images were converted into adaptive CT based on the daily CBCT images using deformable registration (b) clinical workflow for daily dose accumulation using CBCT images to generate aCT images.

Plan was optimized using Eclipse v15.6, the dose calculation used pencil beam convolution superposition (PCS) algorithm, and a 3‐mm setup uncertainty and 3.5% CT Hounsfield Unit to stopping power conversion uncertainty were applied for the robustness plan optimization. The secondary dose calculation was performed using a Monte Carlo (MC) algorithm (Acuros PT, Varian Medical Systems, Palo Alto, CA, USA) and the dose differences are within 3% between PCS and MC. A multiple‐field optimization (MFO) plan with three fields (anterior, right‐anterior oblique, left‐anterior oblique fields) was generated to deliver a uniform dose to the target, in which each side of the target is treated by two fields and the anterior field treats the target on both sides. In the first week of treatment, a quality assurance (QA) CT scan was acquired to validate the blue‐wax bolus regarding its manufacture, fitness to patient face and modeling in the treatment plan. Daily CBCTs were also used to verify the placement of the bolus on the patient. An in‐house developed workflow to assess the daily treatment dose based on daily CBCT images was used to evaluate the inter‐fraction dose accumulation. The adaptive CT images (aCT) using a commercial tool Velocity 4.1 (Varian Medical Systems, Palo Alto, CA, USA),^21^ were generated for each treatment, which considers the anatomical deformation for HU re‐assignment as well as the online rigid matching used during the treatment to account for patient setup variations between the planning CT and the corresponding CBCT.^22^ As shown in Fig. 3, the planning CT images and RT structures were deformably registered to the CBCT images as new aCT, and the treatment plan was forward calculated on the adaptive images to assess target coverage and doses to OARs.

## 3 RESULTS

**FIG. 4:**
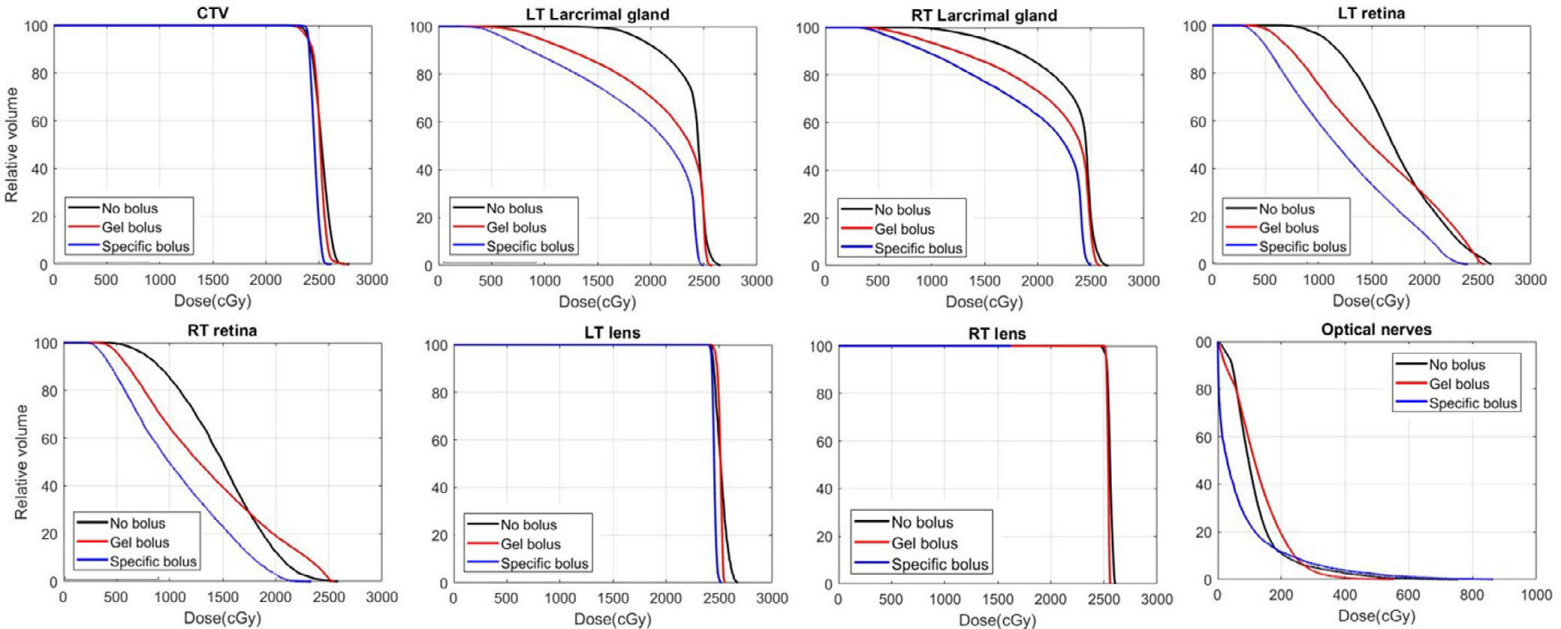
Dose‐volume histogram comparison for no bolus, conventional bolus and patient‐specific bolus plans. In general, bolus plan has reduced organs at risk doses due to the large air gap existing for gel bolus plans. In order to make the plan more robust, the treatment planning system generates additional higher energy layers to compensate for the setup‐induced range uncertainties that result in more overshooting dose for the distal optical nerves with conventional bolus.

**FIG. 5:**
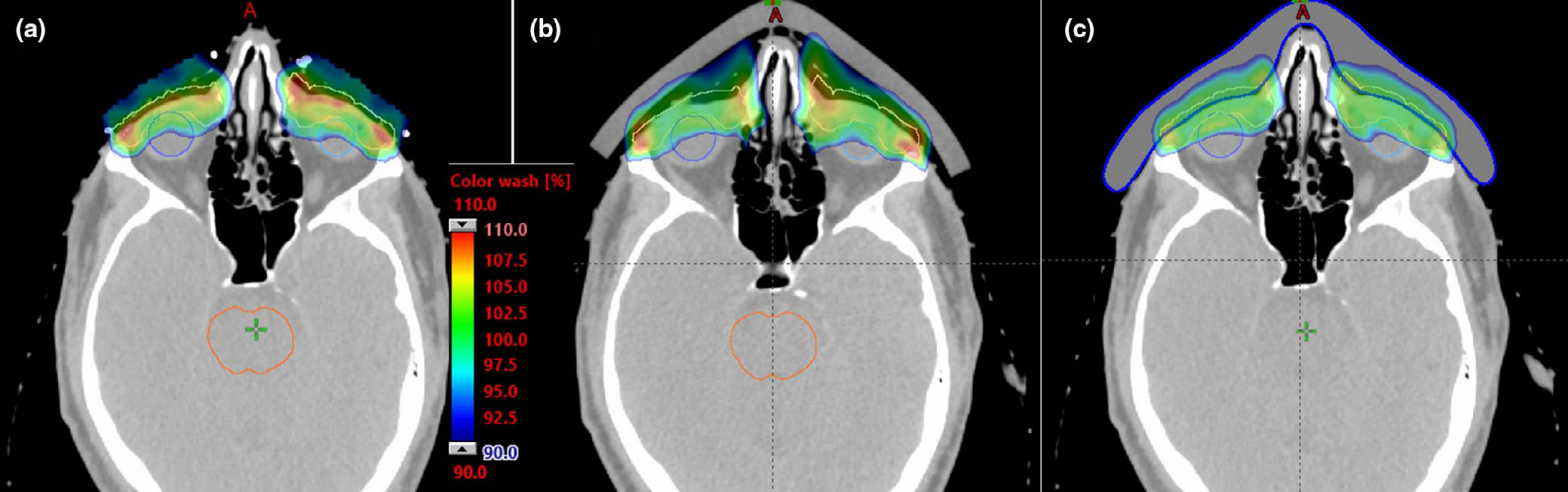
Dose distribution comparison between (a) nonbolus, (b) gel bolus and (c) patient‐specific bolus plans. Color wash shows dose between 90% and 110% of the prescription dose.

The dosimetric characteristics were compared for the nonbolus, gel bolus and patient‐specific bolus plans. The bolus increased proton beam range by adding 2–3 more energy layers of different fields to help achieve a better dose uniformity and improved dose coverage. Figure 4 shows the dose‐volume histogram (DVH) comparisons regarding the dose to the target and critical OARs. The overall plan quality was improved, and the dose uniformity especially was significantly better for the patient‐specific bolus compared to the conventional bolus and nonbolus plans. Figure 5 shows a transversal slice 2D dose distribution, and as seen in Fig. 5(b), the air gap between conventional bolus and skin introduces challenges in achieving uniform dose around the surface due to lack of build‐up materials and also introduces range uncertainties originating from bolus or patient positioning errors. Considering the impact of the air gaps in optimization, the robust optimization allows protons to penetrate deeper, as indicated by the 90% dose color wash in the nasal cavity area, while the blue‐wax bolus plan has less of the 90% dose beyond the intended target. The global maximum dose was considerably reduced by 7% from 116% with conventional gel bolus to 109% with the patient‐specific bolus. Table 1 provides more details of the dose comparison. For instance, the doses were compared between the patient‐specific bolus and the conventional bolus. The bilateral retinal max and mean doses were reduced by 6.0% and 7.7%, respectively, and the bilateral max and mean doses to the lacrimal gland were reduced by 3.0% and 13.9%. The max dose of the lens was reduced by 2.1% from 26.1 Gy to 25.5 Gy. Although being located more posteriorly, the optic nerves still achieved less dose with the patient‐specific bolus compared to the no bolus and conventional bolus plans.

**TABLE 1:** Dosimetry comparison for no bolus, conventional bolus, and blue‐wax bolus plans.

OARs	Blue‐wax Bolus (Gy)		Gel bolus (Gy)		No bolus (Gy)	
	Dmax	Dmean	Dmax	Dmean	Dmax	Dmean
Retina RT	23.4	10.6	22.7	11.7	25.9	23.4
Retina LT	24.1	12.3	25.5	13.0	26.3	24.1
Lacrimal Gland LT	25.0	19.0	25.8	22.3	26.6	25.0
Lacrimal Gland RT	25.1	19.5	25.5	21.7	26.7	25.1
Lens RT	24.9	24.5	25.0	24.4	26.1	24.9
Lens LT	25.2	24.5	26.1	25.2	26.8	25.2
Eye RT	25.5	18.0	26.1	19.1	27.2	25.5
Eye LT	25.4	18.2	25.1	18.4	27.1	25.4
Optic nerves RT	3.3	0.3	3.4	0.4	7.6	3.3
Optic nerves LT	8.6	0.8	5.5	1.3	7.6	8.6

OAR, organs at risk.

**FIG. 6:**
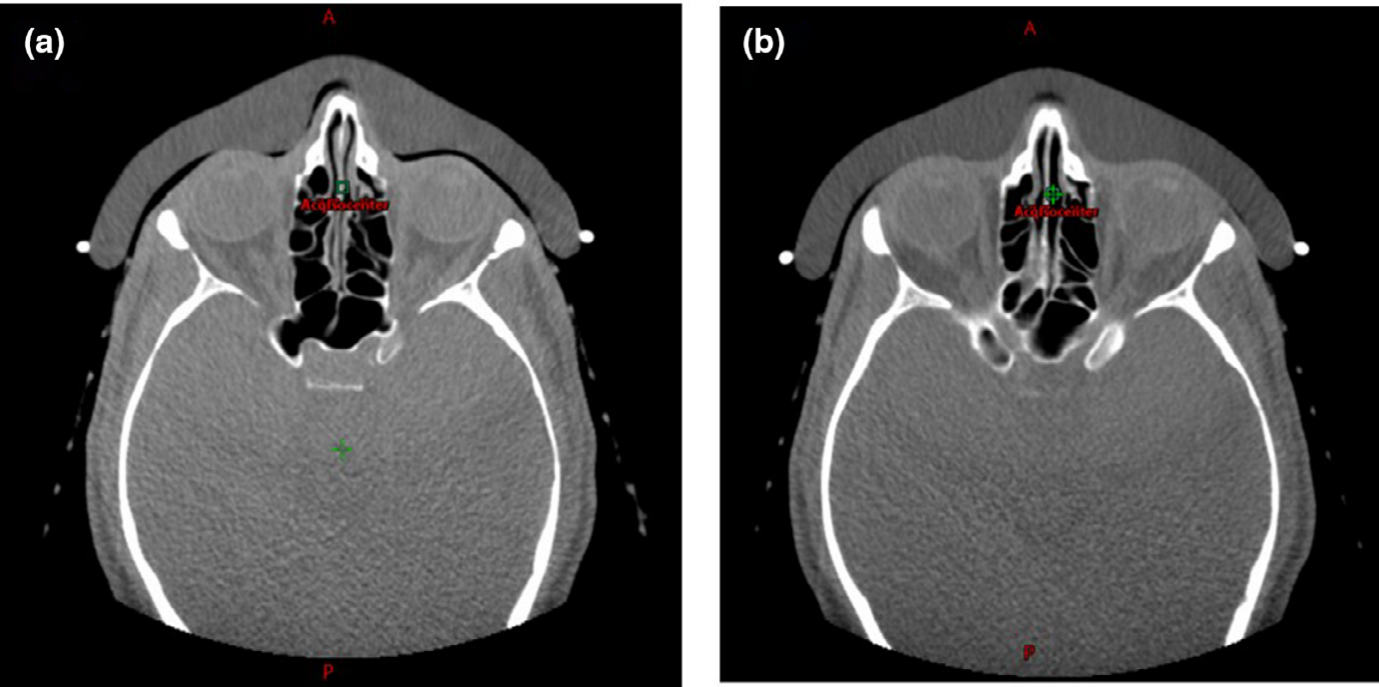
Daily CBCT images of daily setup: (a) a misalignment was identified from an air gap between the bolus and the patient face, (b) the corrected placement of bolus with accuracy of ≤1mm.

**FIG. 7:**
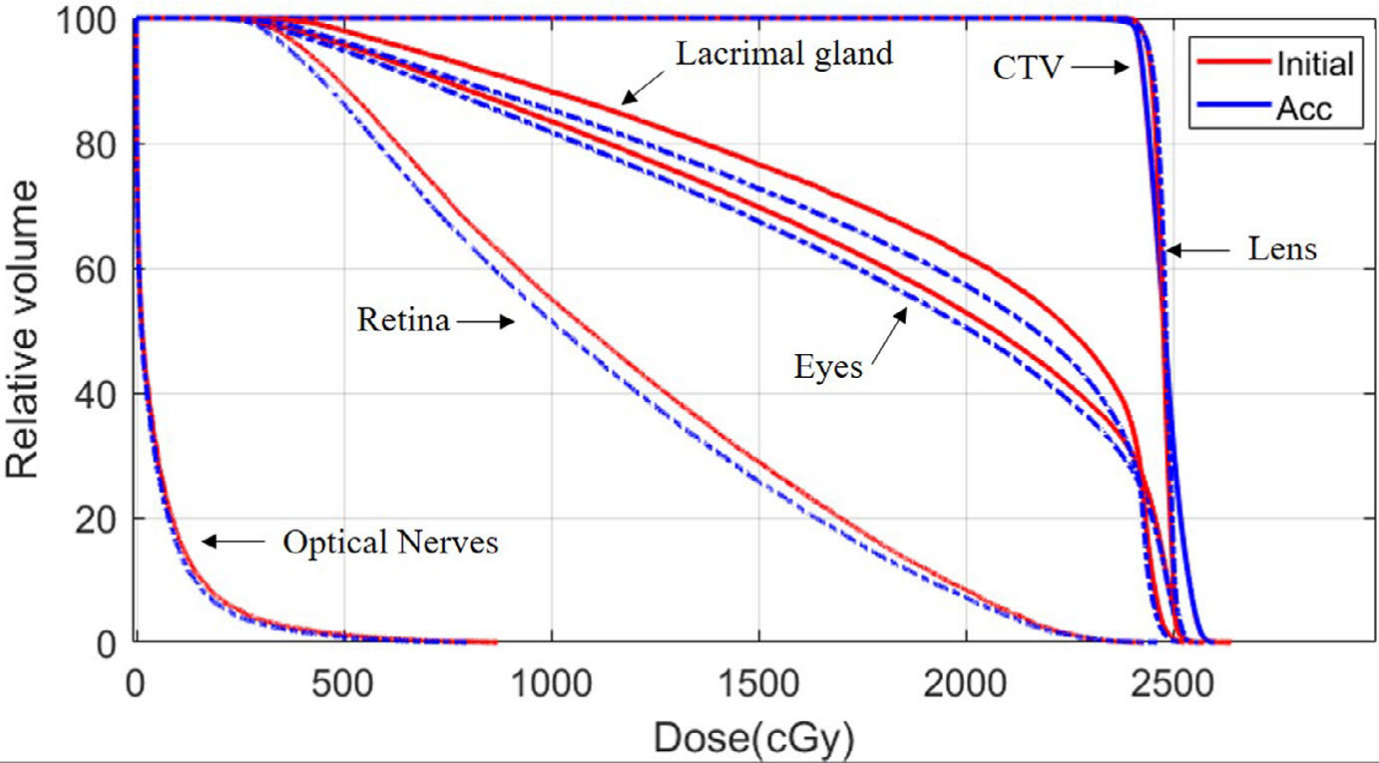
Daily dose comparison between initial plans and daily dose accumulation.

Image‐guided radiation therapy (IGRT) using on‐board kV and CBCT image alignment was performed during daily setup. Two fiducial markers were placed on the blue‐wax bolus and matched with the planning reference images. As shown in Fig. 6(a), the misalignment of the bolus was identified from CBCT images, and a simple re‐placement and re‐image can achieve daily setup accuracy within 1 mm, as seen in Fig. 6(b). The rigid shape and comfortable fit with the patient face helped to maintain accuracy of setups. The inter‐fraction treatment doses were assessed based on the in‐house workflow shown in Fig. 3, and all 12 of the fractional treatment doses were forward calculated using 12‐aCT images. Figure 7 shows in the DVH comparison between initial plan and the accumulated dose using daily CBCT images, the target coverage had no obvious difference and the OARs doses were comparable. The daily dose accumulation also indicated that the patient‐specific bolus plan provided a robust setup and dose distribution using PBS proton.

At last follow‐up, 3 months after completing treatment, the patient was without any clinical or radiographic evidence of disease. No acute or subacute toxicities from proton therapy were noted, and the patient denied any interval conjunctivitis, xerophthalmia, or other treatment‐related adverse events.

## 4 DISCUSSION

The patient‐specific bolus provides an opportunity to maximize advantages of PBS proton to treat very superficial and extremely thin targets immediately adjacent to OARs. The accurate characterization RSP of the bolus materials is one of the essential factors to achieve this goal. As the bolus was contoured in TPS, the CT HU override will be the best method to eliminate the range uncertainties caused by using stoichiometric CT calibration curve.^23^ Using proton beams to measure the RSP should be included as part of the planning and QA procedures.

Factors that contribute to an imperfect match between skin and the bolus are delineation resolution, smoothing of the delineated contour, matching of the bolus to the delineated contour, and deformation of the bolus on the patient.

The misalignment of the patient‐specific bolus with the skin surface will likely result in significant inaccuracies in dose distribution. Sufficient IGRT is important to maintain the treatment position accuracy to reduce any dose uncertainties caused by setup errors. The advancements in deformable image registration tools provide the ability to perform rapid inter‐fraction dose evaluation based on the daily CBCT images. Additionally, fraction dose accumulation has become available for proton systems using on‐board 3D volume image systems. The artifacts of CBCT images and the position difference between planning CT and CBCT images, however, all can contribute uncertainties to the accuracy of deformable vector maps and image quality of aCT. Acquiring high‐quality CBCT images and aligning the patient precisely are essential to make reliable inter‐fraction dose accumulation.

## 5 CONCLUSION

The patient‐specific bolus enables PBS proton delivery to treat challenging cancers more accurately and efficiently. The clinical workflow to adopt patient‐specific bolus in treating periorbital tumors results in greater feasibility, and enhanced patient comfort, while ensuring high‐quality dose distribution. This patient‐specific blue‐wax bolus significantly increased dose uniformity, reduces OAR doses, and maintained consistent setup accuracy within 1 mm. Quality PBS proton treatment for periorbital tumors, and likely other challenging anatomy, can be achieved by using a patient‐specific bolus with robustness on both setup and dosimetry.
